# Emerging Biomarkers in Metabolomics: Advancements in Precision Health and Disease Diagnosis

**DOI:** 10.3390/ijms252313190

**Published:** 2024-12-08

**Authors:** Dang-Khoa Vo, Kieu The Loan Trinh

**Affiliations:** 1 College of Pharmacy, Gachon University, 191 Hambakmoe-ro, Yeonsu-gu, Incheon 21936, Republic of Korea; 2 BioNano Applications Research Center, Gachon University, 1342 Seongnam-daero, Sujeong-gu, Seongnam-si 13120, Gyeonggi-do, Republic of Korea

**Keywords:** biomarkers, metabolomics, cancer, neurodegenerative, diabetes, gut-microbiota, precision health, disease diagnosis, personalized medicine

## Abstract

Metabolomics has come to the fore as an efficient tool in the search for biomarkers that are critical for precision health approaches and improved diagnostics. This review will outline recent advances in biomarker discovery based on metabolomics, focusing on metabolomics biomarkers reported in cancer, neurodegenerative disorders, cardiovascular diseases, and metabolic health. In cancer, metabolomics provides evidence for unique oncometabolites that are important for early disease detection and monitoring of treatment responses. Metabolite profiling for conditions such as neurodegenerative and mental health disorders can offer early diagnosis and mechanisms into the disease especially in Alzheimer’s and Parkinson’s diseases. In addition to these, lipid biomarkers and other metabolites relating to cardiovascular and metabolic disorders are promising for patient stratification and personalized treatment. The gut microbiome and environmental exposure also feature among the influential factors in biomarker discovery because they sculpt individual metabolic profiles, impacting overall health. Further, we discuss technological advances in metabolomics, current clinical applications, and the challenges faced by metabolomics biomarker validation toward precision medicine. Finally, this review discusses future opportunities regarding the integration of metabolomics into routine healthcare to enable preventive and personalized approaches.

## 1. Introduction

Metabolomics is the study of metabolites-small chemical entities taking part in the cellular process of biological systems [1]. Metabolomics research aims at the identification of these small metabolites present in any biological fluid, tissue, or cell that may summarize the metabolic processes in such a way as to suggest both environmental and genetic elements influencing the health of an organism [2]. Unlike genomics or proteomics, which estimate the potential predisposition or levels of proteins, respectively, metabolomics reflects real-time physiological and pathological states to offer dynamic insights into biological processes [3]. The application of metabolomics in health and disease has gained increased prominence. Metabolomics enables the detection of biomarkers for diagnosis, prognosis, and treatment response to diseases based on a specific metabolic signature [4]. Such metabolic profiles occupy a prime position in explaining the pathogenesis of complex diseases such as cancer [5], neurodegenerative disorders [6], cardiovascular diseases [7], and metabolic syndrome [8]. Moreover, metabolomics plays an important role in precision medicine since it makes it possible to identify metabolic differences in individuals that can facilitate personalized treatment modalities [9,10]. Metabolomics, in that view, has now become a powerful complement to personalized medicine, adding new knowledge of the mechanisms behind diseases.

Biomarkers constitute the quantifiable basis of biological states and conditions; therefore, biomarkers are of importance in healthcare, especially in diagnostics, prognosis, and treatment monitoring [11,12]. In diagnostics, biomarkers allow for early and accurate disease diagnosis often before symptoms manifest—a very important issue in such diseases as cancer, where early intervention significantly improves outcomes. For example, specific metabolite profiles can enable the discrimination between disease subtypes or stages with a view to enhancing diagnostic precision and assisting patient stratification [13,14]. Biomarkers may provide prognosis, meaning information about disease development and the course it will take, therefore influencing the outcome of the patient. Identifying markers of disease severity or recurrence can enable healthcare providers to predict disease trajectory and appropriately plan interventions. This especially applies to chronic and complex diseases in which metabolic biomarkers may indicate the risk for complications or rapid development of the disease process. Biomarkers also help in the monitoring of treatments by providing real-time responses in patients [15]. They allow for the examination of efficacy and possible treatment-related side effects, allowing modifications of therapeutic regimens for maximum efficacy with minimum side effects [16]. Through these roles, biomarkers are important in moving precision medicine forward by enabling more personalized, proactive, and effective ways of providing healthcare [17].

This review provides an overview of the general trends of recent findings in the discovery and application of biomarkers based on metabolomics, and their transformative impacts on precision health and personalized medicine. With advancements in technology and analysis capabilities for metabolomics, it is increasingly possible to identify disease biomarkers even for diseases as complex as cancer, neurodegenerative disorders, cardiovascular diseases, and metabolic syndrome. Whereas these biomarkers are of great importance in improving early diagnosis and classification, they also allow a far more nuanced understanding of individual variability in disease progression and treatment response. The aim of this review is thus to review these novel biomarker findings in metabolomics, focusing on the contribution that biomarkers can make to the personalization of healthcare. It has become evident that disease-specific metabolites in a profile and an insight into their pathways bear great potential for further refinement in patient stratification, treatment outcome prediction, and specification of personalized therapeutic strategies. We also discuss the translational development of metabolomics for application in mainstream clinical practice and give special attention to the ability of metabolomics to change healthcare from a one-size-fits-all model to a very personalized model in which any health risk can be identified quickly and treatment can be optimized for maximum effectiveness.

## 2. Cancer Biomarkers in Metabolomics

### 2.1. Metabolic Alterations in Cancer

Cancer cells manifest profound metabolic reprogramming, which allows them to proliferate and survive rapidly and to invade, which is quite different from that in normal cells [18,19]. Such a metabolic shift has been commonly described as the “Warburg effect” [20,21,22]; there is a preference for aerobic glycolysis rather than oxidative phosphorylation, even in the presence of oxygen. As a result of this process, cancer cells produce enormous quantities of lactate that cause changes in the composition of the tumor microenvironment and further promote growth and immune evasion [23]. In addition, cancer cells show disturbed lipid metabolism [24], amino acid dependence [25], and increased nucleotide synthesis [26] to support high cell division rates. Metabolomics has become highly useful for the identification of specific biomarkers attributed to these particular cancerous changes in metabolism. Metabolomics profiling of metabolites in blood, tissue, and other biofluids exposes metabolic signatures associated with cancer that can distinguish malignant cells from normal cells [27]. Certain oncometabolites, such as 2-hydroxyglutarate (2-HG) in gliomas, fumarate, and succinate, serve not only as biomarkers for specific kinds of cancer but also genetic mutations in metabolic enzymes [28,29]. These tumor-specific metabolites shed light on tumor metabolism, unmasking putative therapeutic targets. Furthermore, the biomarkers through metabolomics allow the facilitation of early diagnosis, treatment response, and disease surveillance of cancers to be further advanced toward personalized and precision oncology [30]. This provides clinicians, through metabolomics, the power to understand in real time how cancer cells change their metabolic activity through the progression of a treatment; hence, targeted interventions and personalized therapeutic strategies are opened by these facts.

### 2.2. Key Biomarkers in Oncology

**Figure 1:**
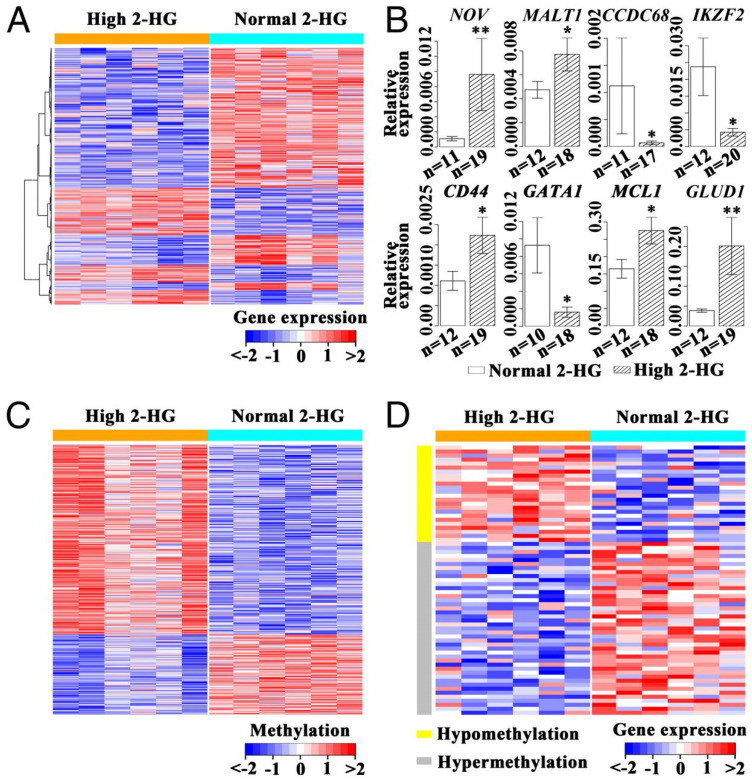
Gene-expression/DNA methylation patterns in leukemia blasts of AML patients with high 2-HG compared with those with normal 2-HG. (**A**) Display of 1224 genes with significant differences in expression levels between AML cases with high and normal 2-HG. (**B**) Quantitative RT-PCR results for eight genes show different expression levels between AML cases with high and normal 2-HG. * *p* < 0.05, ** *p* < 0.01. (**C**) DNA segments of 203 genes with significant differences in methylation levels between AML cases with high or normal 2-HG. (**D**) Display of 67 genes with correlation between modification of DNA methylation patterns and changes in expression levels in the high 2-HG group compared with the normal 2-HG group. Copyright PNAS (2013) [34].

Biomarkers, especially oncometabolites, are of critical importance in cancer diagnostics, prognosis, and therapeutic targeting [31]. Oncometabolites are metabolic intermediates that, at aberrant levels, drive neoplastic disease and, largely, are the result of mutations to metabolic enzymes [32,33]. Generally, these biomarkers have immense value not only in the identification of cancers but also in predicting disease outcomes and treatment response. A well-recognized oncometabolite is 2-HG, resulting from mutations in the isocitrate dehydrogenase 1 (IDH1) and 2 (IDH2) enzymes in gliomas and acute myeloid leukemia (AML) (Figure 1) [34]. 

High levels of 2-HG act both as a diagnostic biomarker and as a prognostic biomarker, since 2-HG, at high levels, is a factor impairing cellular differentiation, hence causing tumorigenesis [35]. For instance, Dinardo et al. underline the diagnostic and prognostic usefulness of 2-HG in AML [36]. This study established that significantly high serum 2-HG levels were associated with IDH1 and IDH2 mutations and demonstrated high diagnostic sensitivity and specificity: 86.9% and 90.7%, respectively, at a threshold of 700 ng/mL. In addition, serum 2-HG levels were associated with clinical outcomes, with higher levels linked to increased tumor burden and worse prognosis. The findings indicate that 2-HG holds the promise of a non-invasive biomarker for the identification of IDH mutations and the follow-up monitoring of disease development and treatment response in patients with AML. It would also be relevant, with regard to biomarkers research, to provide some methodological or standard details regarding their clinical validation and implementation. Moreover, critical attention by Miller et al. is given to mutations of IDH and their metabolite, 2-HG, in glioma diagnosis and treatment [37]. Detectable with non-invasive methods, elevated levels of 2-HG act as a trustworthy biomarker for IDH-mutant gliomas. In clinical respects, IDH mutations provide better survival rates than are otherwise noted, distinguishing them from gliomas with wild-type IDH. Moreover, targeted therapies against such mutations hold the promise of changing the tumor ecosystem to improve therapeutics. These results highlight the importance of IDH mutations in advancing diagnosis and treatments for glioma with precision.

Another important biomarker involves succinate, which accumulates due to mutations in an enzyme, succinate dehydrogenase (SDH), in certain types of paragangliomas, and pheochromocytomas [38,39]. Similarly, the accumulation of fumarate, due to fumarate hydratase (FH) mutations, happens in hereditary leiomyomatosis and renal cell carcinoma [40,41]. These metabolites act as “oncometabolites” in driving DNA methylation [42] and hypoxia-like responses [43] and, therefore, have roles in the identification of hereditary cancer syndromes and in evaluating cancer risks [44,45]. Moreover, changes in lipid metabolism metabolites, including phosphocholine, are being identified as biomarkers in both breast and prostate cancers [46,47]. A high level of phosphocholine and other lipid-related metabolites is indicative of tumor growth and can deliver diagnostic information regarding cancer subtypes. These critical biomarkers, on the one hand, support early diagnosis but on the other hand, they provide important information regarding tumor behavior and make personalized prognosis possible, hence targeted therapies. Biomarker identification and their clinical application keep expanding under continuous scientific advancement. These improve precision oncology and allow treatments that are really tailored to an individual’s metabolic profile.

### 2.3. Clinical Applications and Challenges of Cancer Biomarkers in Metabolomics

Biomarker metabolomics holds great potential for the early diagnosis of cancer, patient stratification, and follow-up on treatment responses [48,49]. The early stages of tumors could be identified by the clinician through tumor-specific metabolic profiles, often well before symptoms present. For example, oncometabolites such as 2-HG are now in clinical use for diagnosing gliomas and assessing mutation status [50,51], while altered lipid metabolites help in the identification of breast and prostate cancers [52,53]. These biomarkers also provide a basis for the stratification of patients in terms of recurrence or disease progression, as an estimation of probability would provide the clue for appropriate therapeutic interventions [54]. The real-time monitoring of metabolic changes during treatment could also give priority to therapeutic effectiveness to ensure timely adjustments to the treatment that improve outcomes. However, translating metabolomics biomarkers into routine clinical practice has not been devoid of significant challenges. First, the nonuniform protocols for sample collection, processing, and data analysis introduce variability into the process, making reproducibility difficult between studies [55]. In addition, biological variability in the levels of metabolites with age, lifestyle, diet, and microbiome composition complicates the interpretation of metabolomics data, thereby making the task of setting universal thresholds for biomarkers rather daunting [56]. High costs and technical requirements for advanced metabolomics platforms, including mass spectrometry (MS) and nuclear magnetic resonance (NMR), further limit clinical acceptance. Another remaining challenge involves the regulation whereby new biomarkers have to be validated through large-scale, multi-center clinical trials with which to validate their accuracy and reliability [57]. Overcoming these challenges will be crucial to ensure that metabolomics-based biomarkers can make a proper transition from the research domains to clinical applications where it would serve real benefits in the early detection of cancers, personalized treatments, and better patient outcomes.

## 3. Neurodegenerative and Mental Health Biomarkers

### 3.1. Metabolomics in Neurodegenerative Diseases

Metabolomics offers a potent tool in the search for neurodegenerative diseases, enabling the finding of biomarkers linked to disease progression and applied in the processes of diagnosis and treatment monitoring. Diseases such as Alzheimer’s disease (AD), Parkinson’s disease (PD), and amyotrophic lateral sclerosis (ALS) present certain distinct metabolic changes related to the underlying pathophysiological mechanisms in these diseases and, therefore, are helpful in the early stages of detection and differential diagnosis [58]. Biomarkers in Alzheimer’s disease like glutamate, myo-inositol, and phosphatidylcholine relate to neuronal damage, oxidative stress, and inflammation [59,60,61]. For instance, myoinositol level elevation was linked to amyloid plaque deposition, one of the hallmark events of AD [62], and low levels of acetyl-L-carnitine are associated with mitochondrial dysfunction as well as cognitive decline [63]. Other lipid-related metabolites involve certain phospholipids and are studied as biomarkers of the preclinical stages of AD, thus providing the opportunity for early intervention. In the case of Parkinson’s disease, the major metabolomics biomarkers studied include dopamine, homovanillic acid, and uric acid [64]. Central to PD is the reduction in dopamine due to the degeneration of dopaminergic neurons. On the contrary, homovanillic acid, a metabolite of dopamine, is increased and may be a marker of the severity of the disease [65]. Lower levels of uric acid, an antioxidant, were also reported in relation to a higher risk of PD, pointing to oxidative stress as a factor [66]. Biomarkers such as creatine, ascorbate, and various amino acids in ALS reflect disturbances in energy metabolism, oxidative stress, and neuroinflammation [67,68]. Lowered creatine levels indicate deficiencies in cellular energy, while changes in the branched-chain amino acids reflect disturbances in the muscle metabolism of these important nutrients in ALS patients [69,70]. Metabolomics profiling of such biomarkers provides a deeper insight into the mechanisms of neurodegenerative disease and, consequently, may provide early diagnosis and differentiation from other conditions. Moreover, these biomarkers can offer perspectives in the development of targeted therapies since the levels of metabolites can be monitored for assessing the efficacy of treatments and for choosing personalized therapeutic strategies in neurodegenerative care.

### 3.2. Mental Health Biomarkers

**Figure 2:**
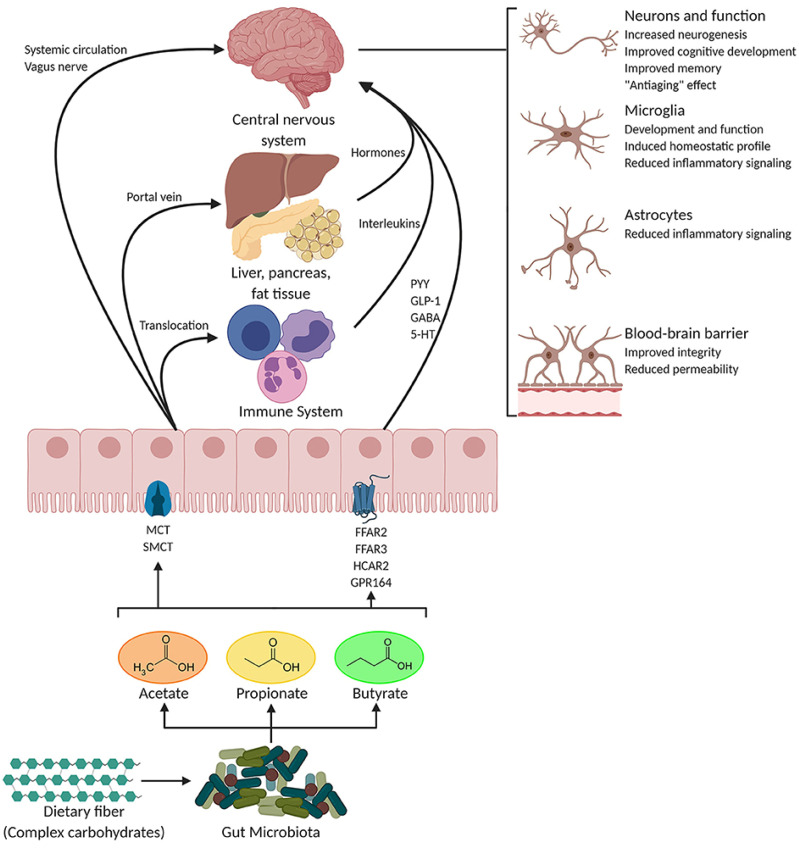
Potential pathways through which SCFAs influence gut–brain communication. Copyright Frontiers Media SA (2020) [78].

Metabolomics has attracted many applications in the field of mental health research, with special regard to the search for biomarkers associated with a variety of psychiatric disorders, including depression, schizophrenia, bipolar disorder, and anxiety [71,72]. The analysis of neurotransmitter pathways and metabolites related to the gut–brain axis holds the promise of indicators for the biochemical basis of diseases related to mental health, thus allowing for more accurate diagnoses and tailor-made therapeutic interventions. Neurotransmitter metabolites are important in psychiatric diseases because their presence or absence directly influences cerebral activity and mood modulation. For example, serotonin, along with its metabolite 5-hydroxyindoleacetic acid (5-HIAA), is extensively studied for mood diseases [73]. Similarly, decreased levels of serotonin and its corresponding metabolites are reportedly found in depression [74], while dysregulations in dopamine and homovanillic acid are linked to schizophrenia and bipolar disorder [75]. Alterations in the level of gamma-aminobutyric acid (GABA), a known inhibitory neurotransmitter, have been associated with symptoms of both anxiety and depression and reflect changes in both stress modulation and mood regulation [76]. In addition, metabolites produced by the gut–brain axis are increasingly being recognized for their involvement in mental health [77]. Short-chain fatty acids (SCFAs), such as butyrate and propionate, result from intestinal microbiota activity and have effects on neuroinflammation, stress responses, and emotional regulation mechanisms (Figure 2) [78]. 

Low levels of SCFAs are suggested to be associated with depression and anxiety because they are implicated in the reduction in inflammation and the preservation of blood–brain barrier integrity [78]. Other gut-derived metabolites, including tryptophan and kynurenine, are associated with immune responses and neurotransmitter synthesis and have been implicated in mood disorders and schizophrenia [79]. These metabolomic biomarkers are important in showing the biochemical underpinnings of psychiatric disorders but, importantly, will allow for a more tailored approach to the treatment of mental health. Such biomarkers, when monitored in a continuous manner, would help health professionals refine diagnostics, tailor therapeutics, and most likely improve patient outcomes by offering more focused interventions.

### 3.3. Potential of Neurodegenerative and Mental Health Biomarkers for Early Diagnosis

Biomarkers based on metabolomics have a great future in the diagnosis and treatment planning of neurological and psychiatric disorders [80]. These biomarkers are expected to help in early intervention through the detection of specific metabolic changes that occur even before the onset of clinical symptoms, thereby bringing improvement in the prognosis by slowing the progression of the disease [81]. A preclinical stage of the disease has, for instance, been observed where increased myo-inositol and changes in phospholipid profiles occur in AD patients, hence their value in early diagnosis [82]. Such biomarkers, if identified early, will enable timely interventions that delay cognitive decline. Clinical studies have shown very promising results using metabolomics as a basis for biomarker discovery for the early diagnostics of neurodegenerative and psychiatric disorders. The lower levels of urates and alpha-synuclein aggregates in CSF have been reported as intriguing early markers for PD [83]. For psychiatric diseases, changes in serotonin and dopamine metabolism have been related to depression and schizophrenia [84,85]. Additionally, elevated levels of kynurenic acid are linked to neuroinflammation and oxidative stress, giving information concerning the early stages of schizophrenia and mood disorders [86,87]. Altered SCFAs derived from the gut–brain axis are showing up as promising biomarkers for mental health disorders, especially depression [88]. In addition, neurofilament light chain levels in ALS and sphingomyelin reductions in multiple sclerosis are reliable early diagnostic markers for these diseases [89]. In anxiety disorders, increased levels of cortisol increase the risk and contribute partially to establishing a diagnosis even before symptoms can completely materialize [90]. Such biomarkers also provide tools not only for early detection but also for applying personalized treatment to improve prognosis and slow down disease progression. 

The early diagnosis of mental health disorders allows clinicians to employ treatment options founded on unique person-centric biochemical profiles that will maximize the effectiveness of an intervention [91]. Eventually, the use of metabolomics-based biomarkers hopefully will translate into a paradigm shift in the way clinicians diagnose illnesses early and select treatments that are tailored to each individual patient, thereby improving patient outcomes by managing both neurodegenerative and psychiatric conditions more effectively. 

## 4. Metabolomics Biomarkers for Cardiovascular and Metabolic Diseases

### 4.1. Cardiovascular Biomarkers

**Figure 3:**
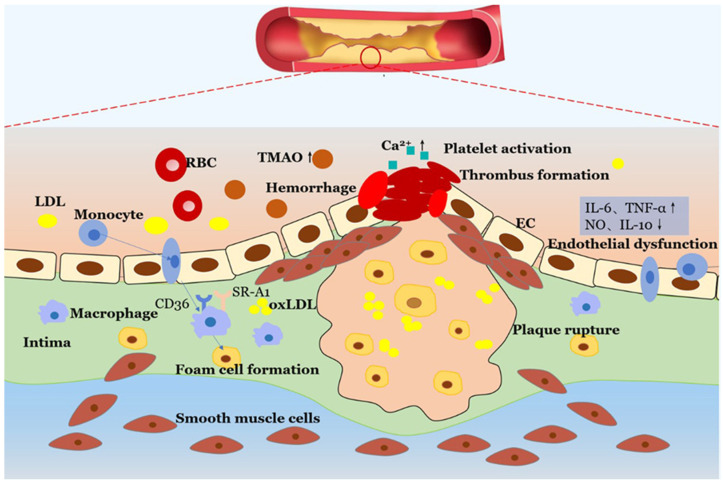
The role of TMAO in atherosclerotic lesion formation and development. The high levels of TMAO in circulation have a crucial role in foam cell formation and endothelial dysfunction; TMAO can activate platelets and promote thrombus generation, making the atherosclerotic plaque vulnerable to rupture. Copyright Wiley (2020) [100].

Lipid biomarkers and other metabolites, revealed by cardiovascular disease metabolomics, characterize important pathophysiological features: inflammation, oxidative stress, and disturbed lipid metabolism [92,93]. These biomarkers have great diagnostic and prognostic values for the identification of persons at risk of cardiovascular diseases (CVD) and help to follow the course of the disease. Lipid biomarkers are central in assessing CVD risk by providing information on changes in lipid metabolism that lead to atherosclerosis and CVD. The most commonly researched lipids are cholesterol esters, phospholipids, and triglycerides, while high levels of LDL cholesterol and high levels of triglycerides are associated with an increased risk for the development of CVD [94]. Furthermore, oxidized phospholipids on lipoproteins serve as markers of oxidative stress and inflammation and predict plaque formation and cardiovascular events [95]. More recently, specific lipid metabolites have surfaced as novel biomarkers in atherosclerosis; the high level of ceramides has been implicated with atherosclerosis and heart failure [96], demonstrating its role in cellular apoptosis and inflammatory pathways. Other non-lipid metabolites, such as trimethylamine N-oxide (TMAO) [97], branched-chain amino acids (BCAA) [98], and betaine [99], are associated with CVD. TMAO, as a product of the gut microbiota metabolism of choline and carnitine, enhances cholesterol deposition in arterial walls, creating a predisposition for atherosclerosis (Figure 3) [100]. 

Higher levels of BCAAs, known to produce metabolic syndrome and insulin resistance, relate to cardiovascular complications [101]. Betaine is a metabolite involved in homocysteine metabolism related to less inflammation and better endothelial function; thus, it may play a protective role against heart diseases [102]. These biomarkers enhance CVD risk prediction and, at the same time, provide possible targets for therapeutic intervention to help in developing more personalized and preventive ways toward cardiovascular health. Characterization of such specific lipid and metabolic biomarkers allows clinicians to better risk-stratify patients, monitor the disease progression, and refine their treatment strategies.

### 4.2. Diabetes and Metabolic Syndrome

**Figure 4:**
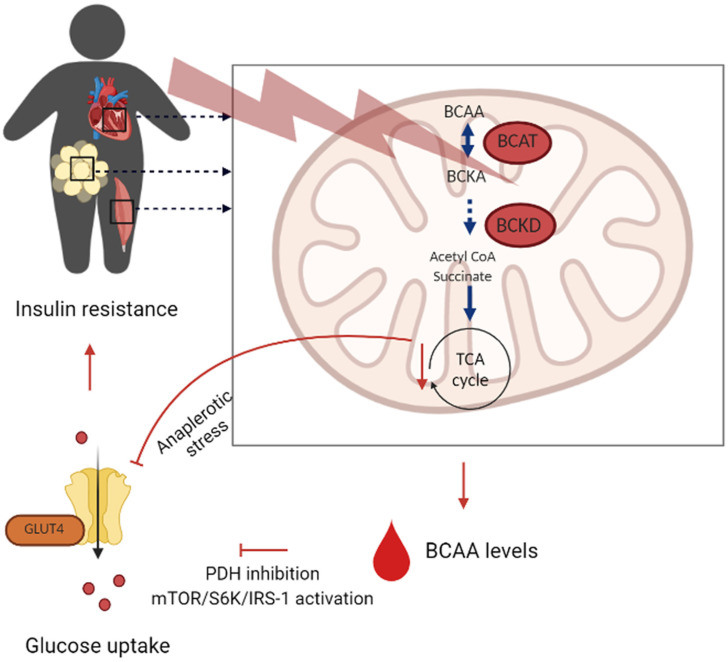
Schematic overview of mechanisms linking BCAA catabolism with insulin resistance. BCAA branched-chain amino acids, mTOR mammalian target of rapamycin complex, S6K ribosomal S6 kinase, IRS-1 insulin receptor substrate-1, PDH pyruvate dehydrogenase complex, GLUT4 glucose transporter type 4. Copyright Nature Publishing Group (2022) [106].

Biomarkers, driven by metabolomics in diabetes and metabolic syndrome, are informative regarding insulin resistance and obesity, total metabolic dysfunction that is attributed to either of these two diseases [103]. As a matter of fact, biomarkers have been said to facilitate early detection, targeted intervention, and monitoring of therapeutic response hence improving metabolic health management. Amino acid biomarkers modulate insulin resistance and overall metabolic dysfunction [104]. In many instances, high levels of BCAAs-leucine, isoleucine, and valine-are associated with insulin resistance and are seen in type 2 diabetes (T2D) and metabolic syndrome [105]. BCAA disrupts insulin signaling, leading to impaired glucose uptake in muscle cells and lipid accumulation Figure 4 [106].

Aromatic amino acids, such as phenylalanine and tyrosine, have also been associated with an increased risk of T2D, indicative of the disturbed amino acid metabolism in obesity and insulin resistance [107]. Lipid biomarkers play a central role in diabetes and metabolic syndrome since disturbed lipid metabolism is one of the causes leading to insulin resistance and systemic inflammation. Free fatty acids (FFAs) are present in high levels of obesity and further increase insulin resistance and inflammation [108]. A class of sphingolipids, the ceramides, is implicated in insulin resistance and stands in a very close relationship with lipid-induced cellular stress and apoptosis [109]. High levels of ceramides are predictive of metabolic dysfunction and the development of T2D, hence a potential target of therapy [110]. Other biomarkers include metabolites of glycolytic and tricarboxylic acid cycles. Lactate and pyruvate are the end products of glycolysis that, in metabolic syndrome, have been reported to be high, reflecting impairment in glucose metabolism [111]. Increased levels of tricarboxylic acid (TCA) cycle intermediates, including citrate and succinate signaling mitochondrial dysfunction associated with insulin resistance, have been associated with disturbed energy metabolism in obesity [112]. Moreover, biomarkers such as this are important for the early diagnosis of metabolic disorders; they also bear importance regarding personalized treatment modalities. Such surveillance of specific metabolic signatures can identify not only people with high risks for diabetes and metabolic syndrome but also provide the clinician with the possibility of disease progression tracking in a timely manner to fine-tune their intervention in order to optimize metabolic health.

## 5. Role of Gut Microbiome and Environmental Exposures in Biomarker Discovery

### 5.1. Gut Microbiota-Derived Biomarkers

Gut microbiota metabolites, especially SCFAs and bile acids are becoming increasingly imperative as biomarkers of metabolic and immune health [113]. The metabolites from the microbiome-derived, produced by fermented nutritional ingredients, act as essential modulators in maintaining metabolic homeostasis and manipulating immune responses, hence governing general health [114]. SCFAs, including acetate, propionate, and butyrate, are produced by bacteria fermentation of dietary fibers in the gut [115]. SCFAs can be considered signaling molecules interacting with specific G protein-coupled receptors, for example, GPR43 and GPR41, thereby modulating immune cell function while promoting T-cell differentiation toward regulatory T cells. SCFAs, therefore, have anti-inflammatory effects, maintaining the integrity of the intestinal barrier and decreasing systemic inflammation [116,117,118]. Thus, SCFAs are important for keeping the immune system in balance, and dysregulation has been found in several immune-related diseases and disorders such as allergies, autoimmune diseases, and infections. Butyrate, for instance, was demonstrated to influence various immune cells by inducing anti-inflammatory responses and enhancing development into regulatory T-cells [119,120]. It also tightens up gut barrier function, prevents translocation of pathogens and endotoxins into the bloodstream, and reduces systemic inflammation. Reduced SCFA levels are also associated with metabolic syndrome, obesity, and inflammatory diseases, making them useful biomarkers of gut and metabolic health. The second class of metabolites produced by gut microbiota represents the bile acids, which, though synthesized in the liver, are subjected to a wide range of structural modifications in the intestine by gut bacteria to yield secondary bile acids. In addition to their role in facilitating fat digestion, these metabolites are bioactive signaling molecules that influence lipid and glucose metabolism, immune responses, and energy balance. Bile acids are ligands for nuclear receptors, including farnesoid X receptor and membrane-bound G-protein-coupled bile acid receptor 1 (GPBAR1), also known as TGR5 that regulate lipid and glucose homeostasis, and anti-inflammatory pathways [121]. Dysregulation in bile acid profiles is associated with metabolic disorders including insulin resistance, obesity, and non-alcoholic fatty liver disease (NAFLD), suggesting thereby that it could serve as a biomarker for metabolic health [122].

Put together, SCFAs and bile acids are one functional axis that molecularly links the gut microbiome to the host immune function and metabolism and they, therefore, serve as useful biomarkers for monitoring and treating metabolic and immune health. Profiling of these metabolites can offer the clinician insights into the status of a patient’s microbiome, the early signs of metabolic dysfunction, and personalized interventions directed at the restoration of metabolic and immune homeostasis.

### 5.2. Exposure Biomarkers (Exposomics)

**Figure 5:**
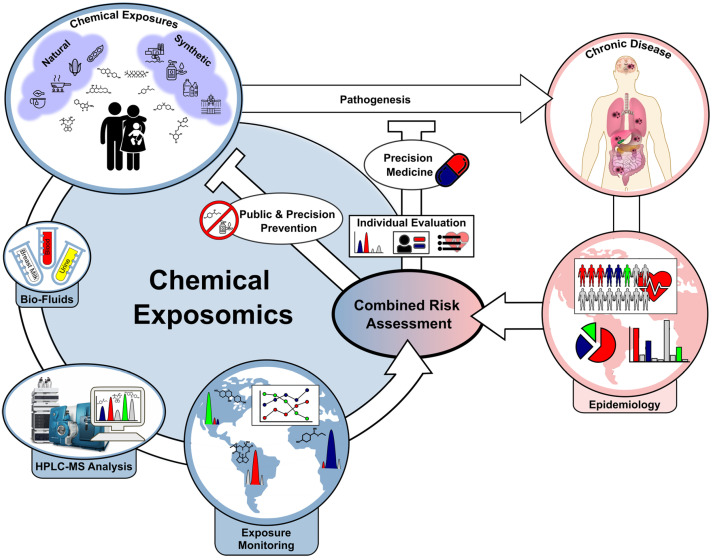
Comprehensive exposomic biomonitoring and combined risk assessment. Schematic illustration depicting the application of a comprehensive exposome approach in an epidemiological setting to improve our understanding of the impact of chemical co-exposure on chronic disease. The prevention of disease will be aided by informed policy decisions and individualized precision medicine. Copyright Nature Publishing Group (2022) [130].

Exposomics, or the study of environmental exposures and their impacts on the metabolome, is helping illustrate the role diet, pollutants, lifestyle factors, and other environmental elements have in defining human health [123,124]. The measurement of these biomarkers of exposure—that is, exposomics—informs investigators about how these external factors influence the metabolisms and, consequently, diseases and population health [125]. Among the numerous classes of exposures, those coming from diet are arguably the most valuable contributors to metabolic profiles. Conversely, for instance, diets high in processed food, added sugars, and unhealthy fats are characterized by the expression of biomarkers of inflammation, oxidative stress, and insulin resistance—all conditions implicated in metabolic disorders such as obesity and type 2 diabetes. On the contrary, diets with a high intake of fruits, vegetables, and fiber trigger the generation of positive metabolites, like SCFAs, which promote gut health and suppress systemic inflammation [126]. Pollutants include heavy metals, endocrine-disrupting chemicals such as bisphenol A, and air pollutants shown to alter the metabolome [127]. For instance, it has been shown that exposure to polycyclic aromatic hydrocarbons (PAHs) due to air pollution is associated with biomarkers indicative of oxidative stress and inflammation; both these factors increase the susceptibility to cardiovascular and respiratory diseases [128]. Persistent organic pollutants (POPs) disturb the metabolism of lipids and hormones, adding to metabolic syndrome, cancer, and immune dysregulation [129]. The implications for public health are huge, since exposomics enables the identification of those populations that are at risk due to environmental exposure and allows for targeted prevention efforts (Figure 5) [130].

Based on the knowledge of the metabolic fingerprints of certain exposures, politicians and practitioners can support demands for cleaner environments, promote healthier dietary guidelines, and pursue personalized health interventions. Exposomic biomarkers ultimately provide a broad view of how environmental factors sculpt health outcomes, thus allowing early measures at the individual and community levels to reduce disease burdens [131].

### 5.3. Integration of Gut Microbiota-Derived Biomarkers and Exposure Biomarkers into Precision Health

Biomarkers of metabolomics integrated into precision health reflect a better realization of how lifestyle and environmental factors influence health and disease risk for more tailored, proactive care. As a matter of fact, these biomarkers will provide an individual health profile through metabolic response to factors such as diet, pollutants, stress, and physical activity, which will inform not only genetic predisposition but also environmental interactions [132]. Favari et al. (2024) described the large inter-individual variability in metabolism due to genetic, environmental, and lifestyle factors that significantly affect the reliability of metabolomics biomarkers [133]. This indeed suggests a need for such variability in the conduct of biomarker research. Individual metabolic variation may impact both the discovery and validation of biomarkers, hence, challenging general clinical application of research findings. Also, Rattray et al. (2018) discuss the influence of environmental factors, including diet, contaminants, and lifestyle, on the metabolome [134]. Such environmental determinants have the potential to change metabolic profiles and, therefore, introduce complexities in the metabolomics data analysis. The study, therefore, calls for critical consideration of these factors in biomarker research so that the biomarkers can be accurate and universally applicable across different populations. Biomarkers of dietary exposure enable the clinician to understand the consequences of nutrition on metabolic and cardiovascular health [135]. Metabolites of high sugar intake, for example, may reveal risks of obesity, diabetes, and heart disease, along with unhealthy fats and metabolites derived from processed foods [136]. Conversely, metabolites derived from healthy foods-polyphenols and SCFAs-are signals of a healthy diet and a gut microbiome acting in immunity and metabolic balance. Monitoring these biomarkers aids in dietary recommendations that agree with a person’s metabolic needs and lifestyle. Biomarkers are representative of environmental exposure—for example, exposure to pollutants and toxins indicates how that environment could be impairing one’s health. Biomarkers associated with heavy metals, endocrine disruptors, and air quality deliver information on oxidative stress, inflammation, and hormonal disturbances, which might also contribute to the development of cancer, respiratory disorders, and metabolic diseases [137]. These biomarkers of exposure facilitate targeted interventions, either through lifestyle changes or detoxification strategies, that mitigate negative health outcomes from environmental exposures. Integration of these biomarkers within precision health strategies has the dual benefit of supporting early identification of health risks but also marshaling individuals to make informed lifestyle adjustments. This allows a holistic view of health in which interventions are highly personalized and adapted to an individual’s unique metabolic and environmental profile. As precision health is still a developing area, biomarkers through metabolomics hold great promise for changing healthcare to a paradigm that will be preventive, personalized, and acutely sensitive to the subtleties introduced by lifestyle and environmental factors.

## 6. Future Perspectives and Challenges

### 6.1. Technological Advances in Metabolomics

Advances in metabolomics technologies have recently majorly empowered depth in analytics, enabling high-throughput rapid identification of metabolic biomarkers relevant to health and disease. Advanced high-throughput screening innovations, new mass spectrometry methodologies, and artificial intelligence (AI)-driven data analysis now also permit profiling of the very complex metabolic networks with unprecedented accuracy and at speeds not previously imaginable. These high-throughput screening techniques alter the scope of metabolomics by analyzing thousands of metabolites from one sample [138]. High-throughput screening, combined with the latest developments in ultra-performance liquid chromatography and tandem mass spectrometry, enables faster and more complete metabolic profiling with increased sensitivity [139]. Such a facility is of utmost importance when large studies are carried out when biomarkers need to be discovered, and there is real-time monitoring of metabolic responses against any intervention. Large datasets that result from metabolomics are increasingly subject to AI and machine learning algorithms. AI enhances biomarker discovery since it clearly identifies small patterns and relationships between metabolites that are hard to notice with a manual process [140]. To be precise, machine learning models allow the classification of disease states, disease risk prediction, and even modeling of personalized responses to treatment according to metabolic signatures. These tools smoothen the processing of data, enhance the accuracy of biomarkers, and allow personalized health assessment because they integrate metabolomics data into other omics layers—for example, genomics and proteomics [141]. This pushes the boundaries of precision medicine and makes metabolomics much more powerful for understanding complex biological processes, thereby enabling translation into clinical applications for metabolomics-based biomarkers. It is because, as the technology in the world keeps improving and developing, metabolomics will become more critical in tailored medicine to inform better medical interventions and decisions by doctors with more precision.

### 6.2. Challenges in Biomarker Validation

For any research in biomarkers, rigorous methodologies must be followed to affirm their clinical validity and utility. An important initial step is the analytical validation, focusing on the accuracy, precision, sensitivity, and specificity of the biomarker assay. The definition of key performance parameters should also include the limit of detection and reproducibility under variable conditions. This is succeeded by clinical validation, where one links the biomarker to distinct diseases or clinical outcomes. Extensive research in heterogeneous populations is important for the validation of diagnostic precision, predictive significance, or prognostic applicability of the biomarker. Standardization is the foundation for consistency in various studies by demanding uniformity in the mode of sample collection, processing, and analysis. So, international standards from different accredited organizations, like the Clinical and Laboratory Standards Institute, will be adopted to reduce variance. In terms of regulatory approval, regulatory agencies like the FDA or EMA, complete information must be provided about safety and efficacy, along with all data regarding its use for clinical decision-making [142,143,144]. The final step to successful clinical implementation is integrating the biomarker into the diagnostic workflow, which would include clear guidelines, clinician training, and analysis of cost-effectiveness to ensure access and practical use. All these methodologies put together are very important in transferring biomarkers from research into routine clinical use and maximizing their potential to improve precision medicine and patient care.

Despite this promising clinical utility of metabolomics biomarkers, some issues remain regarding their validation. The challenge with regard to standardization, reproducibility, and clinical validation still impairs the translation of these biomarkers into common healthcare practice [145]. Primarily, metabolomics methodologies are not standardized. The lack of standardized protocols for collecting, processing, and analyzing samples may lead to differences in the measurement and identification of metabolites [146]. Some factors that will determine the results of such studies are the nature of analytical techniques used, for example, NMR and LC-MS; time of sample collection; and storage conditions [147]. There is, therefore, a need to have standardization across different laboratories so that consistency can be maintained in metabolite profiling and studies can then be compared. Without such standardization, confirmation of results in different populations and settings becomes problematic, limiting therefore the validity of identified biomarkers [148]. Another critical issue is reproducibility. Biomarkers that seem promising in initial studies often fail to reproduce consistent results in larger cohorts or different populations [149]. This inconsistency could be due to variations in the genetic background, lifestyle, or environmental influences of the study participants. Such rigorous validation has to be ensured across diverse populations and clinical settings, with detailed metadata collection considering confounding variables. 

Clinical validation itself has a host of challenges. For a biomarker to be clinically useful, it needs to not only demonstrate statistical significance but also translate to clinically useful outcomes in the form of improved diagnosis, prognosis, or treatment response. This will require large longitudinal studies that link biomarker levels with specific health outcomes. In addition, regulatory approval of biomarkers for clinical use entails strict requirements regarding evidence of their reliability, clinical utility, and cost-effectiveness. This validation process can be very time- and cost-intensive, which has led to the delay in using potential biomarkers in clinical practice. Overcoming these hurdles is critical to the effective integration of metabolomics biomarkers into precision medicine. Harmonization of methodology continues, improvement of reproducibility, and well-designed clinical validation studies will be necessary to set up these biomarkers as valuable tools in disease prevention, diagnosis, and personalized treatment strategies.

While offering tremendous potential, metabolomics faces numerous constraints and controversial questions. The lack of consensus about the coherence of biomarkers from study to study, mainly arising from population diversity, environmental influences, and variations in analytical procedures, remains one major challenge. For instance, many multi-omics studies of irritable bowel syndrome still did not find uniform characteristics of gut microbiota [150]. The complexity would be in establishing a disease-specific metabolic signature. Differences in sample handling, experimental design, and data interpretation may also make the results from studies on the same disease contradictory, thereby leading to issues of reproducibility. The complex interaction between host and microbial metabolic processes adds even more layers of complexity to the identification of unique biomarkers. Such limitations emphasize the requirements for standardized methodologies, more diverse participant groups, and integrative strategies accounting for confounding variables. These challenges must be addressed in order to translate metabolomics findings into robust clinical applications.

### 6.3. Clinical Utility and Future Directions

Metabolomics-based biomarkers are extremely useful in enhancing the evaluation of risks and personalized management approaches for a wide array of diseases, such as cancer, cardiovascular disease, neurodegenerative diseases, diabetes, and psychiatric illnesses. These biomarkers will inform the clinician of an individual’s detailed metabolic profile, which in turn leads to early diagnosis, stratification of the patient, and monitoring of the patient’s therapeutic response. The metabolic markers are evaluated to enable health professionals to better know who is at risk for specific diseases and take an earlier approach before symptoms appear [151]. By integrating these biomarkers into clinical practice, personalized screening, and risk prediction become feasible; this will shift healthcare from a reactive model to one that is preventive and anticipatory [152]. Personalized management allows metabolomics biomarkers to personalize the treatment strategy according to the individual’s unique metabolic needs. Biomarkers of lipid origin are a good example in cardiovascular disease, pointing the way to lipid-lowering therapies [153]. Neurotransmitter metabolites in mental health help with the customization of psychiatric treatments. Real-time monitoring of metabolic changes during treatment allows the clinician to assess treatment effectiveness and allow protocol adjustments as necessary, further refining the therapeutic approach [154]. Further areas of development will thus include the extension of biomarker validation through large-scale clinical studies, further biomarker standardization, and integration of metabolomics with other omics data in pursuit of a more complete view of patient health. Developments in high-throughput mass spectrometry and associated informatics will continue to develop the sensitivity and precision of biomarker discovery [155]. It is obvious that metabolomics can reform healthcare toward increasingly tailored, proactive, and patient-centered outcomes by refining risk assessment and enabling very personalized management strategies.

The Metabolomics Standards Initiative (MSI) has emphasized the need to ensure robust experimental design in metabolomics studies at a minimum of five biological replicates [156]. This benchmark ensures that variability is reduced and thus the highest reliability of the metabolomics data is achieved. However, this decision would be better if it were guided by power analysis, representing the estimated sample size based on biological differences and variability expected or present in a dataset. Commonly, metabolomic studies are underpowered and, therefore, carry the risk of producing unreliable or non-reproducible results, hence limiting the validity of the biomarkers identified using such studies. The required sample sizes will be larger for more variable biological material and in the conditions of increasing study variables such as diverse populations and environmental conditions. In consequence, balancing the resource constraints with the required statistical power to detect meaningful effects, though possibly small, is in the interest of researchers trying to avoid underestimating metabolomic data complexity.

### 6.4. Opportunities for Precision Medicine

With the integration of metabolomics into precision medicine, various opportunities emerge that advance the cause of personalized and preventive healthcare. In this connection, at least several directions point toward changes that metabolomics might bring about in improving patient outcomes and eventually redefining health management strategies. In personalized health profiles, metabolomics enables us to create complete metabolic profiles of individuals, indicating their unique biochemical responses to environmental exposures, dietary patterns, and lifestyle choices [157]. Thus, with the knowledge of each of the profiles of metabolism, health professionals are able to institute interventions according to each particular metabolic condition for better efficacy of treatment and minimal adverse drug events. Early disease diagnosis inherent in its capability to detect specific biomarkers corresponding to various diseases is early detection and intervention [158]. Metabolomics can identify metabolic changes that occur long before clinical manifestation has started, thus paving the way for early intervention in serious and deadly diseases like cancer, diabetes, and cardiovascular disease. Early intervention greatly helps in improving prognosis and reducing disease burden. For preventive healthcare strategies, metabolomics offers a view into the metabolic risk factors of diseases that aid in the development of personalized preventative strategies [159]. This gives healthcare providers a clue about those individuals whose metabolic profile is at higher risk of developing certain diseases. It then offers an opportunity for the implementation of lifestyle modification, dietary intervention, and/or focused screening programs, which can bring down the incidence of chronic diseases. Integration between metabolomics, genomics, proteomics, and transcriptomics will probably be part of precision medicine in the future. Accordingly, the multi-omics approach can give a more holistic view of health that could lead to deep insights into disease mechanisms and allow the identification of novel therapeutic targets [160]. 

Integration of the multi-omics approaches will bring great promise to propel precision medicine, since omics approaches such as genomics, transcriptomics, proteomics, and metabolomics reveal the holistic understanding of a complex biological system [161,162]. Technologically, this synergy permits unraveling intricate interactions of genetic, transcriptional, and metabolic processes that had previously been associated with an illness, therefore opening entirely new dimensions in biomarkers and therapeutic targets. For instance, the combination of metabolomics with genomics could explain how genetic variations impinge on metabolic pathways and hence enhance disease prediction as well as personalized interventions [163]. However, challenges still need to be overcome, including the integration of large, multidimensional datasets, the establishment of standardized analytical pipelines, and the interpretation of cross-omics correlations in view of complex biology. The cost and level of expertise required for multi-omics studies are also generally only feasible at present with highly funded projects. Of even more importance to the fulfillment of multi-omics approaches will be the advances in bioinformatics and machine learning, including collaborative research addressing those challenges in precision health.

In terms of real-time monitoring and management, wearable technologies and point-of-care metabolomics devices in development enable real-time monitoring of metabolic changes [164]. This enables modification of treatment in real time because of variability in individual responses, thus making care more personalized, with increased adherence to the respective therapy. Data from metabolomics, combined with AI and machine learning, will further increase predictive modeling and risk assessment. These technologies analyze complex datasets to supply patterns and correlations that drive clinical decision-making and result in more accurate diagnoses and treatment approaches [165]. On a more general scale of public health implications, this information extracted through metabolomics can be used in public health policies and programs. Understanding metabolic effects attributable to environmental exposures and lifestyle factors at the population level would further public health policy and strategies toward minimizing risk while promoting healthier communities [166]. Precisely, metabolomics holds great promise for pushing the future of precision medicine. This approach, via metabolic profiling, enables us to advance toward personalized, preventive medicine and health for better health outcomes and quality of life in diverse populations.

## 7. Conclusions

New biomarkers keep on coming in metabolomics, redefining the precision of health and diagnostics of disease, enabling insight into the biochemical etiology of various diseases. These make metabolomics a potentially valuable platform for early detection, prognosis, and implementation of personalized therapeutic strategies based on a comprehensive understanding of metabolic change due to disease and environmental factors. The biomarkers identified through metabolic profiling have great potential in cancers, cardiovascular diseases, neurodegenerative disorders, and metabolic syndromes for improving diagnosis and enabling targeted interventions. Nonetheless, there is still a long way to go before the biomarkers can actually make it to the clinic. Interindividual variability, environmental influences, and data complexity in metabolomics need to be taken into serious consideration to make the biomarkers reliable and reproducible in diverse populations. For that, there needs to be a rigid methodology in biomarker validation with large cohorts and considerations for confounding factors. Advanced technologies in high-throughput screening, artificial intelligence, and integrative data analysis should be brought in to enhance the accuracy and clinical utility of metabolomics. Such challenges notwithstanding, the potential for revolutionizing healthcare is big for metabolomics. This field is being pushed continuously toward personal therapies by biomarker-dedicated research and translation to clinical practice, depending on characteristics of metabolic profiles and away from uniform treatment. Only through continuous research and collaboration among clinicians, researchers, and technologists will it be possible to overcome many of the challenges currently in existence so that the translation of biomarkers from metabolomics into clinical practices can occur for better patient outcomes and chart the course forward for precision medicine.

## Data Availability

Not applicable.
